# Accurate Energy Consumption Modeling of IEEE 802.15.4e TSCH Using Dual-BandOpenMote Hardware

**DOI:** 10.3390/s18020437

**Published:** 2018-02-02

**Authors:** Glenn Daneels, Esteban Municio, Bruno Van de Velde, Glenn Ergeerts, Maarten Weyn, Steven Latré, Jeroen Famaey

**Affiliations:** IDLab Research Group, University of Antwerp—imec, 2000 Antwerp, Belgium; esteban.municio@uantwerpen.be (E.M.); bruno.vandevelde@student.uantwerpen.be (B.V.d.V.); glenn.ergeerts@uantwerpen.be (G.E.); maarten.weyn@uantwerpen.be (M.W.); steven.latre@uantwerpen.be (S.L.); jeroen.famaey@uantwerpen.be (J.F.)

**Keywords:** IEEE 802.15.4e, TSCH, energy modeling, OpenMote

## Abstract

The Time-Slotted Channel Hopping (TSCH) mode of the IEEE 802.15.4e amendment aims to improve reliability and energy efficiency in industrial and other challenging Internet-of-Things (IoT) environments. This paper presents an accurate and up-to-date energy consumption model for devices using this IEEE 802.15.4e TSCH mode. The model identifies all network-related CPU and radio state changes, thus providing a precise representation of the device behavior and an accurate prediction of its energy consumption. Moreover, energy measurements were performed with a dual-band OpenMote device, running the OpenWSN firmware. This allows the model to be used for devices using 2.4 GHz, as well as 868 MHz. Using these measurements, several network simulations were conducted to observe the TSCH energy consumption effects in end-to-end communication for both frequency bands. Experimental verification of the model shows that it accurately models the consumption for all possible packet sizes and that the calculated consumption on average differs less than 3% from the measured consumption. This deviation includes measurement inaccuracies and the variations of the guard time. As such, the proposed model is very suitable for accurate energy consumption modeling of TSCH networks.

## 1. Introduction

The well-known IoT paradigm is comprised numerous devices that connect to the Internet and contribute to world-wide interconnectivity. Low energy consumption is generally expected of the connected devices, while at the same time being confronted with challenges such as a low expected manufacturing cost, mobility while being connected and deployment in often difficult-to-reach places. This makes minimizing the energy consumption, while still fulfilling strict reliability demands, one of the major challenges of IoT communications.

To achieve high reliability with minimal power consumption, many research works have been conducted on MAC protocols featuring these requirements [1]. An important development was the IEEE 802.15.4 MAC layer and more specifically the IEEE 802.15.4e MAC amendment that proposed the TSCH mode. TSCH-enabled networks achieve a reliability of 99.999% with minimal power consumption, proving to be a promising solution for wireless industrial networks. TSCH uses channel hopping to improve reliability by minimizing the effects of external interference and multi-path fading. In order to limit the power consumption, it uses a time-synchronized schedule that tells a node exactly when to send and receive data and thus avoids wasting energy during contention periods and idle listening. The deterministic nature of TSCH scheduling allows for precise modeling of the energy consumption as has been done in the work by Vilajosana et al. [2]. Such a model allows for a detailed energy analysis of new TSCH scheduling functions or new protocols on top of the TSCH MAC layer (e.g., routing protocols), during simulated or real-world experiments.

In this paper, we propose a novel, more accurate and up-to-date energy consumption model for the IEEE 802.15.4e TSCH mode. It consists of two main contributions. First is a new energy consumption model, based on the work by Vilajosana et al. [2], built from the ground up. It includes a more up-to-date and elaborate set of time slots and states, while using state-of-the-art IoT hardware and firmware. Additionally, the model is extended to support variable packet sizes, a feature absent in the previous work, that allows for a more accurate energy consumption analysis for all packet sizes. As a second contribution, new state durations and state energy consumption measurements are presented for both the 868 MHz and 2.4 GHz frequency bands, using state-of-the-art OpenMote hardware [3,4]. Moreover, we experimentally verify the accuracy of our model by comparing the calculated values for both the 868 MHz and 2.4 GHz band to the measured values and compare the model to that of Vilajosana. Finally, the new measurements are used to analyze the end-to-end performance of a TSCH network using the official IPv6 over the TSCH mode of IEEE 802.15.4e (6TiSCH) simulator [5].

The remainder of this article is structured as follows. In Section 2, we elaborate on TSCH and related work on energy modeling. Subsequently, Section 3 introduces the model itself. In Section 4, the measurement methodology is discussed and the measurement values are presented. Afterwards, the proposed model is evaluated in Section 5 by analyzing calculated values and measured consumption values and comparing the model to a state-of-the-art model. That section also shows the results of the TSCH network simulations to show the energy consumption effects of 868 MHz and 2.4 GHz communication. Finally, Section 6 presents the conclusions of our work.

## 2. Background and Related Work

In this section, we briefly introduce TSCH and the open-source OpenWSN project that is used as firmware. Afterwards, the used OpenMote hardware is discussed. Finally, we compare our work to existing energy consumption models.

### 2.1. Time-Slotted Channel Hopping

In TSCH networks, every node follows a time-synchronized schedule. This schedule instructs every node about exactly what to do and avoids wasting valuable energy. The TSCH schedule is divided into time slots. The duration of a time slot is typically 10 ms or 15 ms and sufficient to transmit a packet of the maximum size of 127 bytes, immediately followed by an optional acknowledgment frame indicating that the packet was successfully received. Multiple time slots are grouped into a slot frame, and the size of a slot frame defines the width of the schedule. These slot frames repeat continuously over time. TSCH also allows one to use multiple frequencies, leading to a two-dimensional matrix of cells. The number of available frequencies actually determines the height of the schedule.

A schedule can contain four possible cell types: TX, RX, shared and off. The first two indicate that the node should send or receive, respectively. Shared cells can be used by any node, and a contention-based back-off mechanism manages the access to it. They are used to synchronize, join and boot up the network [6]. An off cell indicates that the radio of the node should be turned off. The cells in a schedule are updated dynamically by a so-called scheduling function that takes into account the necessary resources to handle the traffic load and prevents wasting resources.

TSCH also uses channel hopping to combat multi-path fading and external interference [7]. This channel hopping depends on the Absolute Sequence Number (ASN) and the number of channels. The exact frequency on which two nodes will communicate is determined by frequency=F((ASN+channelOffset)modnFreq) where *F* is a lookup table containing the set of available channels, channelOffset is the channel offset of the time slot in the schedule and nFreq is the number of available frequencies. The slot frame size (i.e., number of slots in a single slot frame) should be a prime number in order to be sure that every frequency is used.

**Figure 1:**
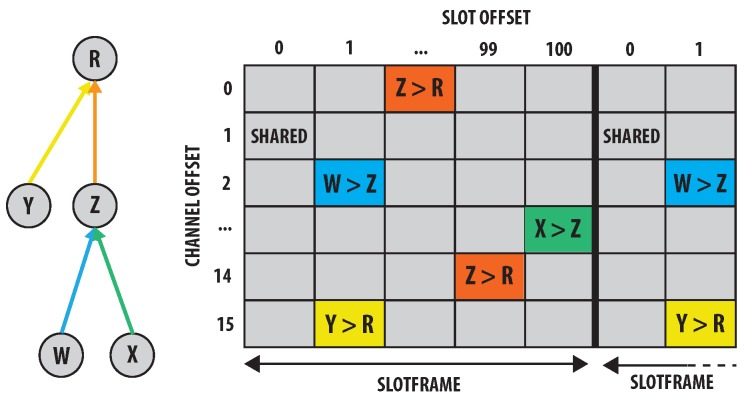
TSCH schedule example.

Figure 1 illustrates an example of a TSCH schedule with a slot frame size of 101 cells and 16 channel offsets. It represents a combination of all schedules of each individual node. Each cell in the schedule represents a specific time slot and channel offset in which directed communication between nodes can be assigned. These assigned cells can either be dedicated to a single transmitter (e.g., from node W to node Z in the cell with a slot offset of one and a channel offset of two), or they can be shared between multiple nodes (e.g., the shared cell with a slot offset of zero and a channel offset of one). All other cells are considered off cells.

This article focuses on TSCH in IEEE 802.15.4e, but the research is also easily transferable to other protocols using TSCH, e.g., WirelessHART and ISA100.11a [8,9].

### 2.2. OpenWSN

**Figure 2:**
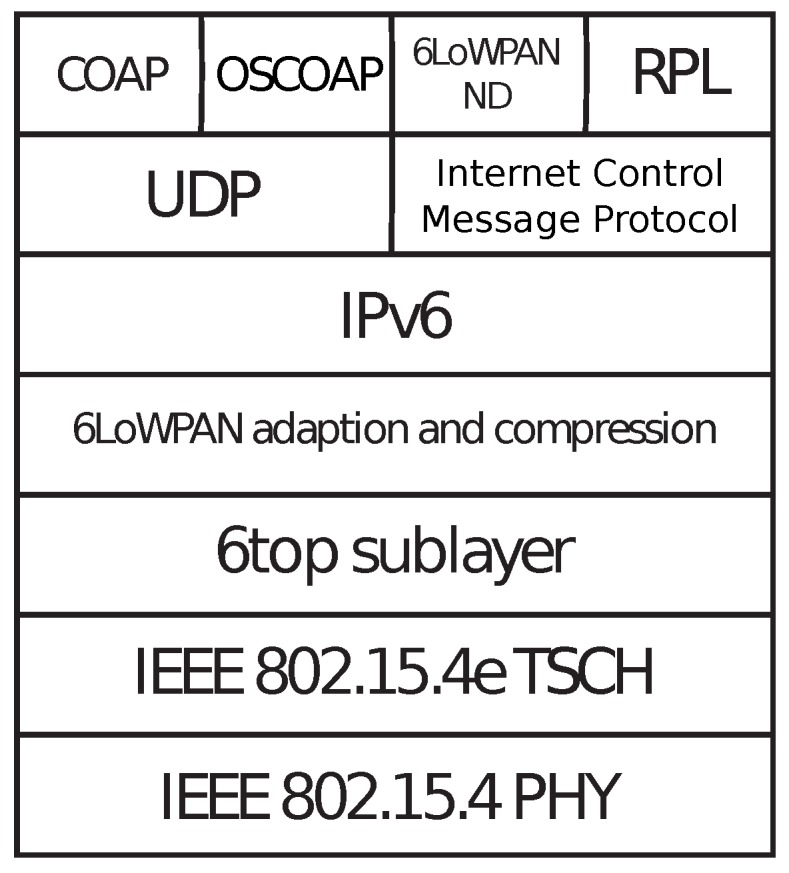
The IPv6 over the TSCH mode of IEEE 802.15.4e (6TiSCH) architecture stack. RPL, Routing Protocol for Low-power and Lossy network.

OpenWSN is an open-source project that implements the IPv6 over the TSCH mode of IEEE 802.15.4e (6TiSCH) architecture [10]. The 6TiSCH network architecture tries to standardize IPv6 on top of the TSCH mode of IEEE 802.15.4e and as such bridge the gap between deterministic industrial networks and traditional IP networks [11]. It aims to provide low latency and high reliability for low-power, critical wireless applications. As such, the OpenWSN firmware provides a complete protocol stack based on IoT standards such as IPv6 over Low-Power Wireless Personal Area Network (6LoWPAN), Routing Protocol for Low-power and Lossy network (RPL) and Constrained Application Protocol (COAP) [12,13,14], as shown in Figure 2. The newest update of the OpenWSN firmware was used when rebuilding and extending the energy model [15]. The hierarchical design of the project makes it relatively easy to port the project to new hardware platforms. Hardware drivers for most common IoT hardware are already available as part of the OpenWSN project itself.

Next to the firmware, useful software such as the OpenVisualizer is also provided. Although the main use of the OpenVisualizer project is to connect the OpenWSN network to the Internet, it also provides the ability to monitor the network. The tool shows the internal state of all the motes that are physically connected to the computer running the OpenVisualizer, e.g., the neighbor table, scheduling table and packet queue. It also has the ability to run simulated motes and to debug the communication with Wireshark [16].

### 2.3. OpenMote Hardware

The measurements presented in this paper are performed using OpenMote, a modular open-hardware ecosystem designed for the industrial IoT [3]. The platform was developed at UC Berkeley and is designed to efficiently implement IoT standards such as 6TiSCH.

OpenMote-CC2538 is the core of the OpenMote hardware ecosystem. It is the most important component, and other components (e.g., the OpenBattery) are considered to be extensions of it. It features a Texas Instruments CC2538 System-on-a-Chip (SoC) that consists of a 32-MHz micro-controller with 32 kB of RAM and an IEEE 802.15.4-compliant 2.4 GHz radio.

The OpenUSB version used in this article has a CC1200 radio chip. Unlike the CC2538, which has a 2.4 GHz radio, the CC1200 is a radio transceiver that operates in the 900-MHz range, e.g., the 868 MHz band in Europe. This allows for longer-range communication between the motes. As OpenUSB only holds the CC1200 radio transceiver, it needs to be connected to OpenMote-CC2538, which holds the microprocessor to control it.

Currently, a new board called OpenMote B is being released. OpenMote B will be a next-generation, dual-band OpenMote device [4]. It provides a dual-radio interface for short- and longer range communication, combined on one board.

### 2.4. TSCH Energy Modeling

As minimizing the energy consumption is one of the major challenges of IoT networks, much research has already been conducted on this topic. Some of the research already focused on TSCH energy modeling.

Some works target specific features in TSCH. De Guglielmo et al. proposed an analytical model of the IEEE 802.15.4e TSCH CSMA-CA algorithm that is used in shared time slots [17]. The authors also observed that the capture effect has a significant impact on the performance of the CSMA-CA algorithm. Papadopoulos et al. investigated the impact of the guard time in TSCH [18]. The authors decreased the guard time duration when motes were closer to their sink and concluded that this results in significant savings in energy consumption without compromising network reliability. While these works only aim at specific TSCH elements, the proposed work provides an energy consumption model for the whole of the IEEE 802.15.4e TSCH mode. Other works such as Juc et al. compared the performance of the TSCH and Deterministic and Synchronous Multichannel Extension (DSME) modes of 802.15.4e [19]. The authors do not propose a model themselves. They observed that TSCH mode tends to consume more energy than DSME mode. This is due to the large fixed guard time in TSCH and because DSME can aggregate multiple acknowledgments and transmit a single group of acknowledgments.

Finally, Vilajosana et al. presented an energy model for TSCH networks, using the OpenMote and OpenWSN for their experimental validation [2]. The values from the model were compared to measurements on the GINA and OpenMote-STM32 platforms. Our paper continues the work of Vilajosana, but explores several differences and improvements. As such, we propose a model with an extra time slot type (i.e., TxDataRxNoAck), provide an extended and a more up-to-date set of states per time slot and extend the model to support variable packet sizes. Furthermore, the OpenWSN firmware has been continuously updated, and the current software version has changed substantially since the version used by Vilajosana in 2013. Finally, by using the OpenMote-CC2538 and OpenUSB board, this paper focuses on state-of-the-art hardware. This allows us to consider the TSCH energy consumption in both the 868 MHz and 2.4 GHz band. To the best of our knowledge, we are the first to do this. We also explicitly look at the difference in power consumption between using a SoC and the case with a separate micro-controller and radio chip. All the steps in developing the model are explained in detail, allowing it to be used for different types of hardware by simply changing the measured consumption values.

## 3. TSCH Energy Model

In this section, the proposed TSCH energy model is introduced. First, all types of time slots are discussed, followed by a more detailed examination of the states in these time slots. Afterwards, the time slot energy model is presented. Finally, we explain how the slot model could be adapted for use with different hardware. The implementation of the proposed model can be found in [20].

### 3.1. TSCH Time Slots

A TSCH schedule can contain different types of time slots, i.e., cell types, to indicate that a node should transmit, listen or put its radio to sleep. In IEEE 802.15.4e, seven different types of time slots can be identified:

- TxDataRxAck: The mote sends a frame during this time slot and receives an ACK when the data have been received successfully.
- TxData: The mote sends a frame during this time slot, but does not expect an ACK (e.g., broadcast or multicast frames such as RPL Destination Oriented Directed Acyclic Graph Information Object (DIO) messages).
- RxDataTxAck: The mote listens and receives a frame in this time slot and replies with an ACK to indicate that it successfully received the frame.
- RxData: The mote listens and receives a frame in this time slot, but no ACK is sent (e.g., broadcast or multi-cast frames).
- RxIdle: The mote listens, but does not receive a frame in this time slot.
- Sleep: The mote does not transmit or receive during this time slot.
- TxDataRxNoAck: The mote sends a frame and expects an ACK, but no ACK is received. This could be caused by a collision of the data frame.

**Figure 3:**
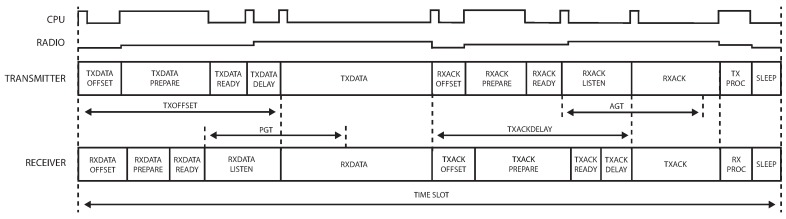
States in TxDataRxAck (transmitter) and RxDataTxAck (receiver) time slots, together with the CPU and radio activity in the TxDataRxAck time slot.

The proposed model divides each time slot into different states. Figure 3 illustrates this and presents a general overview of the activity of a transmitter and receiver during a TxDataRxAck time slot and RxDataTxAck time slot, respectively. Some of the states seen in Figure 3 consist of two parts: one part where the CPU is active and one part where the CPU is sleeping. These two parts are considered as separate states in our model. The state of the radio in our model only changes at moments when the CPU state changes. This is a simplification as in the real world, the radio state changes slightly before or after this moment, typically while the CPU is active.

The remainder of this section explains the TxDataRxAck time slot in full detail. The other slots are modeled similarly, and we limit the discussion to highlighting the differences with the TxDataRxAck slot.

#### 3.1.1. Time Slot TxDataRxAck

The different states of the TxDataRxAck time slot are shown in Figure 3, and Table 1 lists the exact CPU and radio states at each moment. As can be seen in the table, the CPU has two states, i.e., Sleep and Active, while the radio has five states, i.e., Sleep, Idle, Listen, Transmit (TX) and Receive (RX).

At the beginning of each time slot, the CPU wakes up and performs the tasks required for any slot. This includes incrementing the ASN and scheduling the next state depending on the type of the slot. The CPU then sleeps again during TxDataOffset until the moment the radio is needed.

During TxDataPrepare, the radio wakes up, the channel is set and the bytes to transmit are loaded into the radio. The duration of this state is variable, mainly because the time necessary to load the bytes depends on the frame size. Since this state always starts at the same offset and has a variable duration, there is some time left between the TxDataPrepare and the actual transmission. During this TxDataReady state, the radio is in Idle mode, while waiting until it is time to transmit. To minimize the energy consumption of the mote, the duration of the TxDataReady state should thus be as short as possible.

The first byte behind the Start-of-Frame Delimiter (SFD) has to be transmitted exactly TxOffset ms after the start of the time slot. In order to do so, the time required to switch the radio from Idle to TX mode has to be taken into account. The duration of the TxDataDelay equals the time between the TX command being sent to the radio and the moment the SFD has been transmitted.

After the RxAckOffset that follows where the mote sleeps, the RxAckPrepare state then prepares the radio again by waking it up and setting the correct channel. Any time less than the maximum duration of RxAckPrepare is then spent in the RxAckReady state.

The ACK is transmitted TxAckDelay ms after the end of the TxData state. Because the clocks of the transmitting and receiving node may not be perfectly synchronized, the ACK might arrive slightly earlier or later than expected. The radio is thus turned on at the start of the RxAckListen instead of just in time for the data. If no ACK is received during the Acknowledgment Guard Time (AGT) period, the mote turns off the radio and considers the transmission failed. The duration of the AGT is defined as 1000 μs in OpenWSN. When the clocks between the motes are perfectly synchronized, the RxAckListen state has a duration of AGT/2 plus the time to change the radio from Idle mode to RX mode (which is considered to be instantaneous in OpenWSN).

During the TxProc state, the ACK is read from the radio and the transmission is considered successful when the ACK is valid. The mote also synchronizes its clock based on the offset between TxAckDelay and the actual data reception time, if the ACK came from its parent in the network routing graph. For the remaining part of the time slot, both the CPU and radio are in Sleep mode.

#### 3.1.2. Time Slot RxDataTxAck

This time slot can be considered the opposite of the TxDataRxAck. The states to handle the data in TxDataRxAck are found in handling the ACK in RxDataTxAck and vice versa. All states of the RxDataTxAck time slot can be found in Table 2.

The guard time for the data is however larger than the AGT that is used for ACKs. The Packet Guard Time (PGT) determines how long the radio listens for the data before the radio is turned off. When no data are received during the PGT period, we classify the time slot as RxIdle instead of RxDataTxAck. In OpenWSN, the PGT is defined as 2600 μs.

#### 3.1.3. Time Slot TxData and RxData

When no ACKs are required (e.g., for broadcasts), only the first half of the time slot is used. During the TxData and RxData slots, the mote sleeps once the data have been transmitted or received. The states for both TxData and RxData are shown in Table 3 and Table 4, respectively.

#### 3.1.4. Time Slot RxIdle

When the transmitter has no data to send, the slot that could have been a TxDataRxAck becomes a Sleep slot. However, on the receiver side, a different type of slot is needed to represent the behavior of the node: the RxIdle slot occurs when the receiver expects data, but does not receive anything. The states of RxIdle are shown in Table 5. The behavior of RxIdle is not an error; it simply means that a slot was reserved, but the transmitter did not have any data to send at that moment.

#### 3.1.5. Time Slot Sleep

In time slots where no data have to be transmitted or received, the node sleeps during the whole duration of the slot. The CPU of the node only briefly wakes up at the start of the slot, e.g., to increment the ASN. The states of the Sleep time slot are shown in Table 6.

#### 3.1.6. Time Slot TxDataRxNoAck

There are many error states in OpenWSN. The code would go into an error state when, for example, the radio remains active too long or when the prepare state lasts longer than the maximum allowed duration. It is unlikely that the code would end up in most of these error states unless there is a configuration issue. However, there is one error state that is likely to occur eventually: a missing ACK. In the TxDataRxAck slot, data are transmitted and an ACK is received, but in the slot that we refer to as TxDataRxNoAck, the ACK is expected, but not received. In this case, the node stays in the RxAckListen state during the AGT period and does not enter the RxAck state. After the AGT period, the radio goes to sleep during the TxProc and Sleep state, as can be seen in Table 7.

### 3.2. TSCH Energy Consumption Model

Having identified all states per time slot, the model for the charge drawn during a time slot can be constructed. The resulting charge (in coulombs) drawn from the battery during a slot, $Q_{Slot}$, is represented by:

(1) $$Q_{Slot}=\sum_{State\in Slot}\Delta t_{State}\times I_{State}$$

with $\Delta t_{State}$ and $I_{State}$ the state duration and current drawn in each state, respectively. The unit of the duration is milliseconds (ms), while the unit of the current is milliamperes (mA), meaning that the unit of the resulting charge is microcoulombs (μC). This can be used to calculate the total charge drawn for each of the slot types discussed in Section 3.1.

Subsequently, the model previously proposed by Vilajosana et al. [2] can be employed to calculate the total charge drawn across a slot frame. This in turn can be used to compute the lifetime of a mote. That model, however, has one major shortcoming. It does not consider the actual packet size when calculating the charge drawn by a slot. Instead, it takes the consumed charge values for the maximum packet size and scales those linearly based on the actual packet size:

(2) $$Q_{slot}^{N_{sent}}=\frac{N_{sent}}{max_{PktSize}}\times Q_{slot}^{max_{PktSize}}$$

With $N_{sent}$ the number of bytes being sent in the packet and $max_{PktSize}$ the maximum packet size for which measurements were performed. However, this leads to highly inaccurate estimates, especially for small packet sizes, as the duration of most states with the slot is independent of the packet size. In contrast, we propose a more accurate estimation of the charge drawn in a slot $Q_{slot}^{N_{sent}}$, based on actual measurements with different packet sizes. This is achieved by expressing the duration of each state that depends on the packet size, as a linear function of the packet size, rather than a fixed value for the maximum packet size. This is elaborated on in Section 4.

### 3.3. Different Hardware Support

Since the model has an elaborate set of parameters, adapting the model to different hardware while maintaining an equal level of accuracy is a burdensome task. However, at the cost of a slight decrease in accuracy, the model can easily be simplified in order to apply it to different hardware. For example, one can set the duration of short states to zero (e.g., TxDataDelayStart and RxAckOffsetStart) and only update the states that have the most impact on consumption. Alternatively, the duration can be estimated instead of measured as most durations will be very similar to the ones presented in this article. Furthermore, the consumption of the CPU and radio does not have to be measured: these values can be found in the data sheet of the manufacturer. The resulting model will be slightly less accurate, but no or only a few additional measurements have to be made to use this model to simulate the charge drawn by other TSCH hardware.

## 4. Measurements

This section first presents the setup used to measure the duration and energy consumption of each state of each slot type. Afterwards, the measurements of the time slot state durations are discussed together with how the duration values are affected by the packet size. Finally, the consumption of each device state is presented with a detailed discussion for each of the two evaluated radios.

### 4.1. Methodology

In this section, the necessary adaptations to the OpenWSN firmware, that allowed performing the measurements, are briefly explained. Additionally, the two measurement setups for both the state duration and energy consumption measurements are discussed.

#### 4.1.1. Firmware Changes

To perform valid measurements, the firmware code that toggles debug pins and LEDs on the OpenUSB board was disabled. Furthermore, the serial communication code was also completely disabled because even when the OpenUSB is not connected to a computer, the code would still try to output data, unnecessarily increasing power consumption.

In order to prepare the 2.4 GHz driver for the measurements, only small adaptations had to be made to the original firmware. The firmware for the 868 MHz driver however required additional implementation effort, as there were no working drivers for the CC1200 radio chip on the OpenUSB. Based on a branch of the official OpenWSN repository [21], we implemented a working CC1200 radio driver, which can be found in [15].

#### 4.1.2. State Duration Measurements

**Figure 4:**
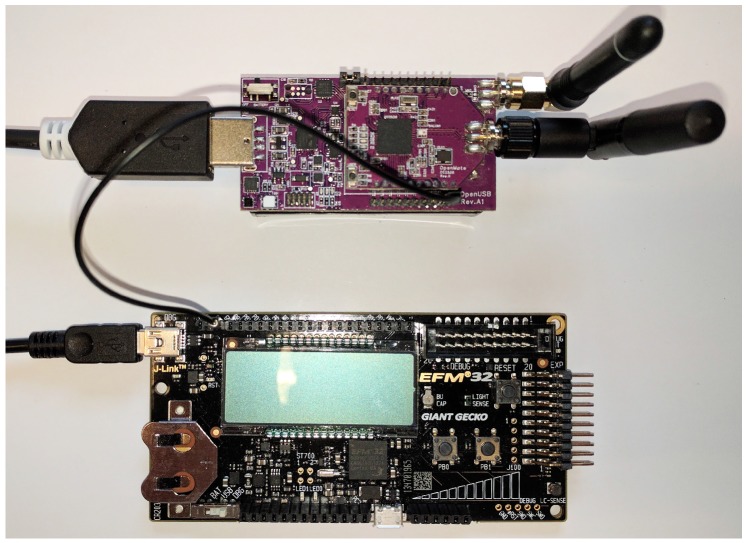
Setup used to measure state durations with a connection from the PB9 pin on the Gecko board (bottom) and to the PD2 pin on the OpenUSB (top).

All state duration measurements were done using the EFM32GG-STK3700 Giant Gecko Starter Kit from Silicon Labs [22]. The setup is shown in Figure 4. Using the Gecko board, an OpenUSB pin was connected to pin PB9 of the Gecko board, enabling the Gecko to measure how long the connected OpenUSB pin was made low. The OpenMote firmware would then make the connected pin low at the beginning of the measurement and high at the end of the measurement. The output was sent over Serial Wire Output (SWO) to the console in the proprietary software Simplicity Studio on the connected computer, where post-processing of the duration data was applied [23]. The duration measurements were averaged in case variability between different measurements was noticed.

#### 4.1.3. Energy Consumption Measurements

**Figure 5:**
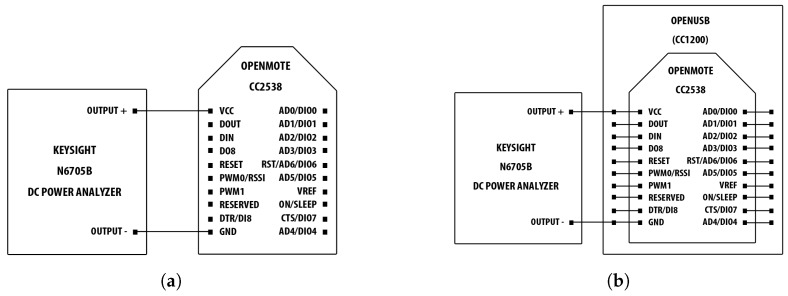
Energy consumption measurement setups: for the 2.4 GHz measurements, only the OpenMote-CC2538 was used, while for the 868 GHz measurements, the power analyzer was connected to the OpenUSB to which the OpenMote-CC2538 was attached. (**a**) 2.4 GHz measurement setup; (**b**) 868 MHz measurement setup.

In order to perform the different energy consumption measurements, a setup different from the Gecko setup, described in Section 4.1.2, needed to be used. As the consumption of the OpenMote hardware happened to exceed 50 mA (i.e., the maximum of the Gecko measuring range), we switched to using the Keysight N6705B DC Power Analyzer [24]. Using the two-wire mode, the Voltage Common Collector (VCC) and Ground (GND) pins of the OpenMote-CC2538, in the 2.4 GHz measurement setup, and of the OpenUSB with the OpenMote-CC2538 attached, in the 868 MHz measurement setup, were connected to the power supply output of the N6705B, which was configured to provide an input voltage of 3.0 V. This is the nominal voltage of two serially-connected AA batteries, which can be used to power an OpenMote via an OpenUSB or OpenBattery module. The measurement setups are shown in Figure 5a,b, for the 2.4 GHz and 868 MHz measurements, respectively. For the 868 MHz measurements, the OpenUSB has to be attached to the OpenMote-CC2538 because the former only holds a CC1200 radio transceiver and needs the microprocessor on the latter to control it.

For most device states, the consumption was averaged over a period of 500 ms. However, some states (e.g., RX and TX states) only last as long as the radio takes to send all bytes. For these states, the average was taken over a period between 3 ms and 4 ms.

### 4.2. Time Slot State Durations

We measured the duration of each state in every time slot where the CPU is active. The durations in which the CPU is sleeping can then be trivially calculated, using the active durations and the timing constants found in OpenWSN firmware. The total length of a time slot was set to 15 ms. The state durations for all time slots are shown in Table 8, Table 9, Table 10, Table 11, Table 12, Table 13 and Table 14.

States do not always have the exact same duration for a variety of reasons. There can be multiple code branches (i.e., different execution paths); the packet size can vary and have an influence; or the duration of an operation can simply be variable (e.g., waking up the CC1200 chip). Therefore, multiple measurements were executed to find a single duration that could be associated with the state.

Changing the mode of the CC1200 radio from Sleep to Idle takes between 246 μs and 343 μs, which causes every state where the radio wakes up to have a variable duration. It only required a few measurements to find that the median for waking up is 268 μs. However, we decided to use the average value instead of the median because it resulted in a slightly more accurate energy consumption prediction. To avoid being susceptible to outliers, the wakeup time was measured over ten thousand times, and an average duration of 273 μs was observed.

For states with multiple code branches, the median value of multiple measurements was chosen. For example, in state TxDataOffsetStart, an Enhanced Beacon (EB) might be sent if and only if there are no data to send. Another example is state TxProc in which the execution path is different when a data packet has no retries left. However, these small variations of the duration only have a limited impact on the total slot consumption.

The durations of states where packets are loaded to and read from the radio were measured before and after the radio was accessed. Afterwards, the communication with the radio for different packet sizes (from 0 bytes–125 bytes with steps of 25 bytes) was measured. Linear interpolation was applied on the measured durations to come up with a formula that fits well to all packet sizes. The difference in durations in states where data are transferred between the radio and CPU (e.g., TxDataPrepare and TxProc) is caused by the way these bytes are transferred: the CC2538 radio is combined with the CPU in one chip, so data can just be copied in/out of memory while the CC1200 needs to use the slower Serial Peripheral Interface (SPI) to transfer data to and from the CPU in the CC2538 chip, resulting in longer durations.

The duration of transmitting and receiving also depends on the packet size. Since the radio has a baud rate of 250 kbps, the time it takes to transmit one bit is 4 μs, which makes the time to transmit a byte 32 μs. To calculate the duration, the amount of transmitted bytes had to be multiplied with 32 μs. The PHY header byte and two-byte Cyclic Redundancy Check (CRC) also have to be included as they are sent with the packet. To verify that this calculation is valid, the time between the start-of-frame interrupt and the end-of-frame interrupt was measured: the average error was only 0.13%.

To model the guard time, we assumed that the clocks are synchronized. Our model thus assumes that the packet always arrives exactly in the center of the guard interval.

### 4.3. Device State Current Consumption

The consumption of the OpenMote-CC2538 connected to the OpenUSB was measured during all possible device states. Since the CPU and radio are the two components responsible for the majority of the current consumption, these device states are all combinations between CPU and radio modes. Instead of measuring the consumption of the CPU and radio separately, we measured the consumption of the entire device. The result is that any current consumption not related to the CPU or radio (e.g., SPI or timers) are measured as part of the CPU usage. This allows for a slightly more accurate prediction of the charge drawn compared to models that ignore these other components.

#### 4.3.1. 2.4 GHz CC2538 Radio

In Table 15, the consumption values of the different device states when using the CC2538 radio, i.e., the 2.4 GHz radio, are shown. The values for the TX state were measured when the transmit power of the radio was set to 0 dBm. When the transmit power was set to 3 dBm, i.e., the current default in OpenWSN, the consumption values of the TX states are 33.04 mA and 29.01 mA, for the CPU in Active and Sleep state, respectively.

The CC2538 radio has an identical consumption of 13.97 mA when the radio is in Sleep or Idle state because the OpenMote-CC2538 consists of both the CPU and radio, and the radio itself does not have a separate Idle or Sleep state. Instead, it has a single Off state for which the consumption was used for both the Sleep and Idle states. Thus, the CC2538 radio has only four states: TX, RX, Listen and Off.

As expected, the difference in the consumption between an active or a sleeping CPU is nearly identical for all radio states: the CPU in active mode consumes on average 3.92 mA more than when being in sleep mode, with a standard deviation of only 0.07 mA.

When switching the CPU of the CC2538 chip to the deeper sleep mode PM2 instead of PM_NOACTION (http://www.ti.com/product/CC2538/datasheet/), while the radio was in the Sleep and Idle state (which are actually both the Off state in the CC2538 radio), the consumption dropped to 1.56 μA.

#### 4.3.2. 868 MHz CC1200 Radio

The device state consumption values when using the CC1200 radio, i.e., 868 MHz, are shown in Table 15. The values for the TX state were measured when the transmit power of the radio was set to 0 dBm. When the transmit power is set to 14 dBm, i.e., the current default in OpenWSN, the consumption of the TX states is 91.94 mA for an active CPU and 88.25 mA for a sleeping CPU. The CC1200 radio Sleep state is the Idle state with the crystal oscillator turned off (http://www.ti.com/product/CC1200/datasheet/). Consumption is expected to be lower when the Sleep state of the CC1200 chip is used or when the CC1200 is turned completely off. As expected, the difference in the consumption between an active.

As expected, the difference in the consumption between an active or a sleeping CPU is nearly identical for all radio states: the CPU in active mode consumes on average 3.81 mA more than when being in sleep mode, with a standard deviation of only 0.15 mA.

When both the CPU and the radio are put in the Sleep state, the consumption is still high. This is caused by the high current consumption of the CPU, which is put in the least possible sleep mode PM_NOACTION. When putting the CPU in a deeper sleep, i.e., the PM2 power mode, while the CC1200 radio is in Sleep and Idle state, the consumption dropped to 0.27 mA and 2.64 mA, respectively.

## 5. Evaluation

In this section, the accuracy of the model is verified. First, the charge drawn per slot type for both radios is calculated and compared to the measured values. Afterwards, the accuracy of the charge drawn during a slot frame is validated using a small-scale test network. The developed packet size-aware model is also compared to a state-of-the-art model to show the accuracy improvement when including the packet size in the model. Finally, using the measured charge consumption values for both frequency bands’ communication, several TSCH network simulations were conducted to observe the energy consumption effects in an end-to-end context.

### 5.1. Slot Charge Consumption

Using the duration and consumption of each state, the charge drawn during each type of slot is calculated using the formula shown in Equation (1). To verify the accuracy of our model, the entire consumption of each type of slot was also measured separately. Table 16 compares the measured and calculated values for both types of radio. Both radios are configured with a transmit power of 0 dBm and a packet size of 127 bytes, i.e., the maximum packet size when including the CRC bytes.

As seen in Table 16, the difference between the measured and calculated values is close to negligible. Among the main contributors to these differences are measurement errors and the variations in guard time duration. In the measured data, the guard time can be smaller or larger than in the calculated data, which assumes perfectly synchronized clocks. On average, the difference is limited to 5.08 μC or 1.55% with a standard deviation of 3.3 μC and a maximum difference of 14.89 μC, which proves the accuracy of our model.

More specifically, for the CC2538, the average relative difference is 0.75%, while for the CC1200 chip, the average relative difference is 2.3%. In the case of the CC1200 chip, the larger relative difference is explained by the fact that a specific device state (e.g., CPU is active and radio is sleeping) does not always result in exactly the same current drawn, which we abstracted in Table 15.

**Figure 6:**
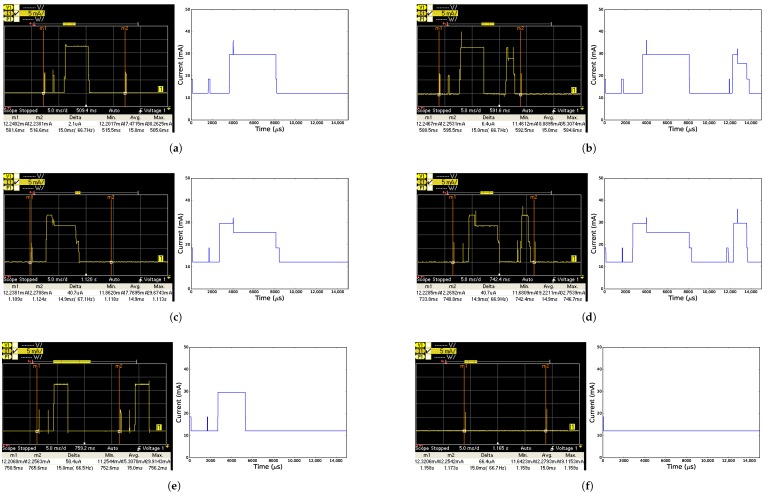
Measured (left, between vertical lines m1 and m2) and modeled (right) current comparison for each time slot when using the CC2538 radio. (**a**) TxData time slot; (**b**) TxDataRxAck time slot; (**c**) RxData time slot; (**d**) RxDataTxAck time slot; (**e**) RxIdle time slot; (**f**) Sleep time slot.

**Figure 7:**
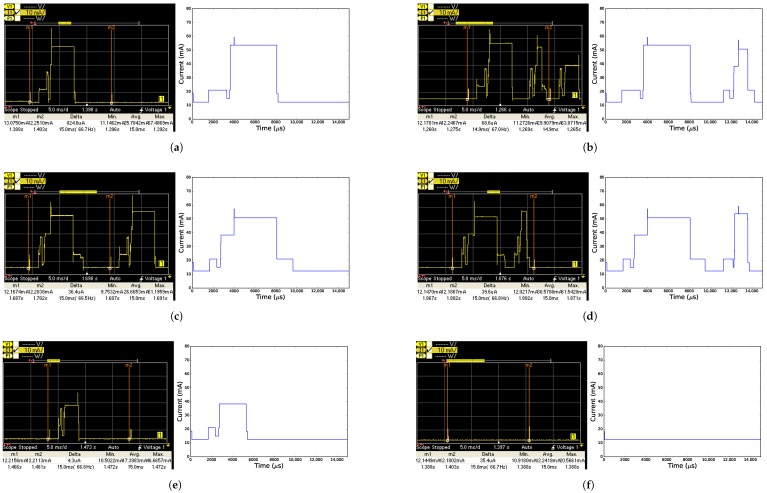
Measured (left, between vertical lines m1 and m2) and modeled (right) current comparison for each time slot when using the CC1200 radio. (**a**) TxData time slot; (**b**) TxDataRxAck time slot; (**c**) RxData time slot; (**d**) RxDataTxAck time slot; (**e**) RxIdle time slot; (**f**) Sleep time slot.

Figure 6 and Figure 7 show the current drawn of all time slots (except for the error time slot TxDataRxNoAck) over time according to both the model and the measurements, when using the CC2538 and CC1200 radio, respectively. For all time slots, the measured graphs and their modeled counterpart look very similar. The peaks on the graphs however do not perfectly match, because the model simplifies certain states. The radio state may be changed while the CPU is active, causing the CPU and radio to be active at the same time, while the model might only consider the radio as active once the CPU goes to sleep. This results in a peak in the measured time slot where there is no peak in the model.

### 5.2. Slot Frame Charge Consumption

When considering the charge consumption in the different time slots, the energy consumption of a slot frame can be calculated. To further verify the accuracy of our model, the calculated slot frame charge consumption of a small-scale, real-world 6TiSCH network is compared with the measured values, for both 868 MHz and 2.4 GHz.

**Figure 8:**
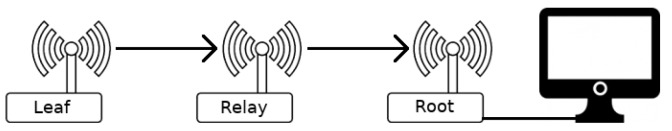
Topology used while comparing the consumption of a slot frame.

The experiment network topology, depicted in Figure 8, used the OpenMote-CC2538 and the OpenMote-CC2538/OpenUSB board combination as hardware nodes for 2.4 GHz and the 868 MHz measurements, respectively. The root node is connected to a computer using OpenVisualizer to monitor the network. The leaf mote was configured to send a packet of 127 bytes (including CRC) every two seconds. The slot frame size was 51 time slots. Since there are 51 slots in a slot frame and every time slot lasts 15 ms, the duration of each slot frame is 765 ms. The first time slot in every slot frame was reserved for management messages, e.g., EBs, RPL DIOs, RPL Destination Advertisement Objects (DAOs) and 6TiSCH Operation Sublayer (6top) messages, but these were not considered. As such, the first time slot in each slot frame is thus considered to be of type RxIdle.

The slot frame of the leaf mote always consists of one RxIdle slot and at least 49 Sleep slots. The last slot will either be of the type TxDataRxAck when there are data to send or another Sleep slot when there are no data. As such, two slot frame types were considered for the leaf node: a slot frame where no data were sent and a slot frame where the packet was sent in a TxDataRxAck slot. The charges consumed in each of these two slot frame types are represented by:

(3) $$\begin{array}{c} Q_{leaf\_Sleep}=Q_{RxIdle}+50\times Q_{Sleep} \end{array}$$

and:

(4) $$\begin{array}{c} Q_{leaf\_TxDataRxAck}=Q_{RxIdle}+Q_{TxDataRxAck}+49\times Q_{Sleep} \end{array}$$

For the relay node, a slot frame was considered where the packet coming from the leaf was received in the first slot of the slot frame; subsequently, the relay forwarded the packet to the root, but no acknowledgment was received, followed by a successful retransmission. As such, there are RxDataTxAck, TxDataRxNoAck and TxDataRxAck slots when a packet was received and forwarded, while the remaining 48 slots are Sleep slots. The charge drawn during the slot frame of the relay node is represented by the following formula:

(5) $$\begin{array}{c} Q_{relay}=Q_{RxDataTxAck}+Q_{TxDataRxNoAck}+Q_{TxDataRxAck}+48\times Q_{Sleep} \end{array}$$

The charge consumption of the root node is not considered as the root device is typically connected to a computer using OpenVisualizer, serving as a gateway to the Internet. Therefore, the root typically does not run on batteries. Additionally, the serial communication between the root and OpenVisualizer cannot be disabled, making the comparison between the measured consumption and the proposed model invalid.

We measured the charge consumed over the length of an entire slot frame for both the leaf and relay node and compared these values to the values calculated using the proposed model and Equations (3)–(5). Table 17 shows the results. On average, the error between the calculated and measured values is lower than 1%. The differences between the CC2538 measured and calculated consumptions, for the leaf and relay nodes, are limited to 1.3% and 1.03%, respectively. For the CC1200 radio, the differences are even slightly smaller: respectively 0.88% and 1.01%. The consumption comparison results again show that our model is accurate, even when measuring across an entire slot frame.

### 5.3. Energy Model Comparison

**Figure 9:**
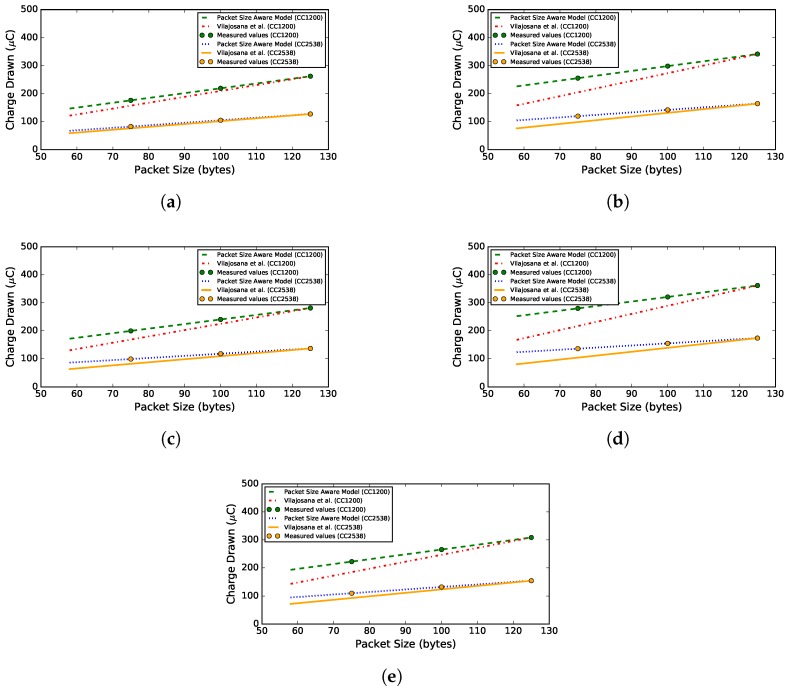
Comparison between the proposed packet size aware model and the model introduced by Vilajosana et al., which linearly scales the charge consumption based on the packet size. (**a**) TxData time slot; (**b**) TxDataRxAck time slot; (**c**) RxData time slot; (**d**) RxDataTxAck time slot; (**e**) TxDataRxNoAck time slot.

In order to indicate the accuracy gain of using the proposed packet size-aware model over the model introduced by Vilajosana et al., these two models are compared in Figure 9. The two models are compared for packet sizes going from 58 bytes, which is the minimum 6TiSCH packet size without additional payload, up to the maximum packet size, i.e., 127 bytes (125 bytes and 2 CRC bytes). The consumption of both models is compared to the packet size-aware model using the exact duration measurements for the packet sizes of 75, 100 and 125 bytes. The device state current values of Table 15 are used for this comparison. In the states where the CPU is sleeping and the radio is sleeping or in idle mode, the PM2 values were preferred over the PM_NOACTION values.

As can be seen in the graphs, the proposed model accurately represents the charge consumption for all packet sizes. The model of Vilajosana et al., however, becomes highly inaccurate especially when the packet size decreases. Looking at a packet size of 75 bytes, the average errors of the Vilajosana et al. model are 32.03 μC (σ=21.05 μC) and 17.05 μC (σ=12.41 μC) for the CC1200 and CC2538 radio, respectively. Of course, for the maximum packet size, both models estimate the consumption correctly.

The reason for this large inaccuracy introduced by the model of Vilajosana et al. is that their approach linearly scales the entire slot consumption. This is not the correct approach, as only the states in which data are transmitted over the radio or copied between the radio and the CPU can be scaled. Since a time slot consists of many more states than only data processing states, those states should not be scaled. Because the proposed model differentiates between the state durations that depend on the packet size and the durations that are independent of the size, as can be seen in Table 8, Table 9, Table 10, Table 11, Table 12, Table 13 and Table 14, it accurately models the slot consumption for different packet sizes.

### 5.4. Frequency Band Consumption Comparison

Using the measured energy consumption values for both 868 MHz and 2.4 GHz, we conducted several TSCH network simulations to analyze the end-to-end network performance and energy consumption at these frequency bands.

#### 5.4.1. Simulation Setup

To perform the experiments, the 6TiSCH simulator is used: an open-source, event-driven Python simulator developed by the 6TiSCH Working Group (WG) [5]. The simulator supports IEEE 802.15.4e TSCH experimentation with straightforward parameter configuration. The configuration parameters for the simulation experiments discussed in this article, are listed in Table 18. To be able to compare the energy consumption for both 868 MHz and 2.4 GHz, we changed the default propagation model of the simulator (i.e., the so-called Pister-hack) to the International Telecommunication Union - Radiocommunications sector (ITU-R) Rural Macro model, which is applicable to both frequency bands [25]. To have a realistic low-power energy consumption comparison between 868 MHz and 2.4 GHz, we re-calculated the charge consumption values of Table 16, using the device state consumption values of Table 15 and adjusted them to make sure the measured CC2538 PM2 power mode consumption value 1.56 μA was used in all states where the CPU was sleeping. When the CPU and radio were sleeping, the Idle state (with the crystal oscillator turned off) consumption value of the CC1200 chip, i.e., the radio Sleep state, was also replaced by the power down Sleep state consumption value of the CC1200 datasheet, which is 0.5 μA (http://www.ti.com/lit/ds/symlink/cc1200.pdf). The resulting slot consumption values are listed in Table 19. The 6TiSCH simulator implementation used for these simulation experiments can be found in [26].

#### 5.4.2. Simulation Results

**Figure 10:**
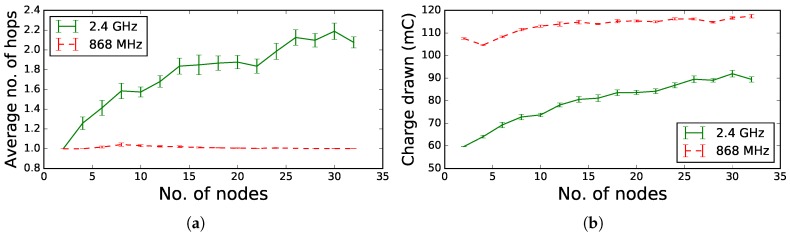
Total consumed charge per node and average hop count for 868 MHz and 2.4 GHz frequency communication in a random topology as a function of the number of nodes. (**a**) Average hop count; (**b**) total energy consumed per node.

In the first TSCH network experiment, the number of nodes in a random topology varies from 2–32 nodes. Figure 10 shows the average hop count and the total energy consumed per node over a period of 300 s. As seen in Figure 10a, for 2.4 GHz, the average hop count increases as the number of nodes in the network increases. For 868 MHz, the hop count stabilizes to one. Communication at 868 MHz is stable over longer ranges than communication at 2.4 GHz, resulting in a lower hop count to reach the root node. For the shorter range 2.4 GHz communication, the increase in consumption is significant when the average hop count increases. Since there are more nodes that have to relay additional packets towards the root, the total consumption per node increases. Figure 10b also clearly shows that the total consumption per node is higher for 868 MHz than for 2.4 GHz as is explained by the absolute slot consumption values in Table 19.

**Figure 11:**
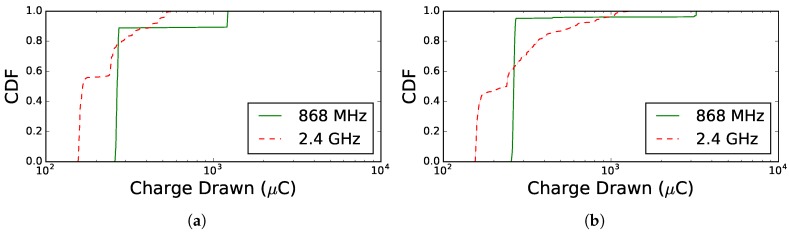
Comparison of charge drawn per cycle per node for 868 MHz and 2.4 GHz frequency communication in a grid topology of nine nodes and 25 nodes. (**a**) Grid topology of nine nodes; (**b**) grid topology of 25 nodes.

In the second TSCH network experiment, the average charge drawn per node per cycle was observed over a period of 300 s. The results are shown in Figure 11, which differentiates between two grid topologies with different sizes: Figure 11a,b shows the Cumulative Distribution Function (CDF) for nine and 25 nodes, respectively. In the network of nine nodes, all eight nodes directly connect to the root node for both 868 MHz and 2.4 GHz. The CDF in Figure 11a shows that almost all of the nodes consume less charge when using 2.4 GHz compared to when using 868 MHz. The difference in consumption is explained by the measured slot consumption values shown in Table 19, which indicates that 2.4 GHz consumes less energy than 868 MHz. However, when looking at nodes between 0.8 and 0.9, we observe that 868 MHz consumes less. The same effect is observed in the results for 25 nodes: 60% of the nodes that use 2.4 GHz consume less than the nodes using 868 MHz. These nodes represent leaf nodes and nodes that do not have to forward many data packets originating from children in the routing graph. Apart from these nodes, there are also other intermediate nodes, as indicated by the 2.4 GHz hop count of 2.49 (σ=1.21) for the grid scenario with 25 nodes, which have to relay many more packets towards the root and consume more energy. When using 868 MHz, the average hop count was 1.01 (σ=0.1), which means that all nodes are directly connected to the root and thus do not relay other packets. For both the grid networks of nine and 25 nodes, the root nodes for 2.4 GHz consume less than those of 868 GHz, which again can be expected by looking at the consumption values in Table 19.

Looking at the absolute energy consumption values for both 868 MHz and 2.4 GHz, an increased energy consumption for all sub-1-GHz communication is expected. However, these simulation results show that due to the longer-range capabilities of sub-1-GHz communication, there can be nodes that consume less energy compared to when using 2.4 GHz communication.

**Figure 12:**
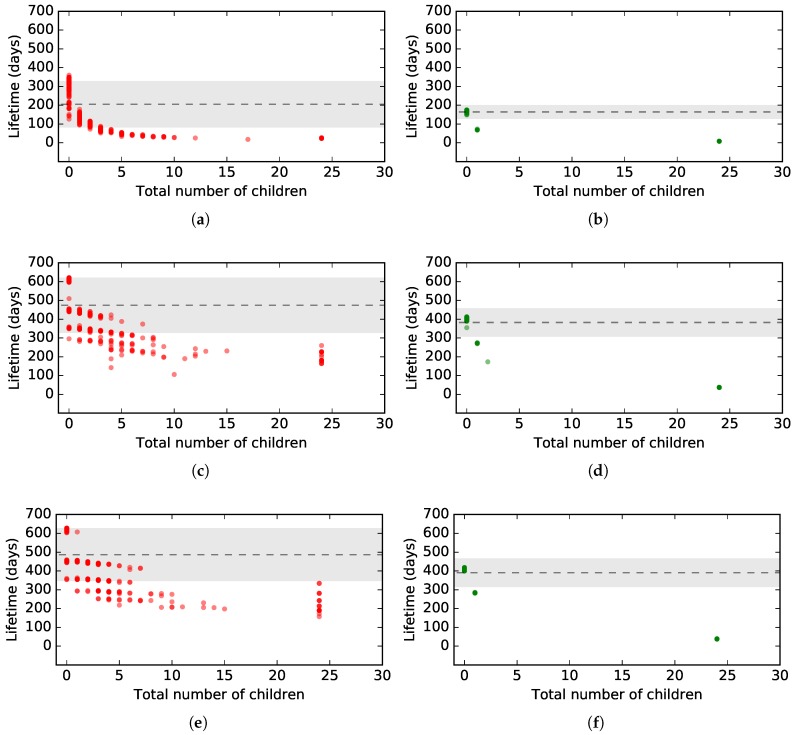
Comparison of the lifetime of a TSCH node, running on two AA batteries, between 2.4 GHz and 868 MHz communication for different packet periods in a grid topology of 25 nodes. (**a**) 1 packet/s, 2.4 GHz; (**b**) 1 packet/s, 868 GHz; (**c**) 1 packet/min, 2.4 GHz; (**d**) 1 packet/min, 868 GHz; (**e**) 1 packet/h, 2.4 GHz; (**f**) 1 packet/h, 868 GHz.

In the third TSCH simulator experiment, we observe the lifetime of all TSCH nodes in a grid of 25 nodes for different packet periods. Each node is assumed to be running on two AA batteries, i.e., a battery capacity of 2000 mA h. Figure 12 shows the results. The total number of children are all children of a node, e.g., the root node will have 24 children. It is clear that in the case of 2.4 GHz communication, there is much more variability in the number of children a node has, compared to when using 868 MHz communication. This is due to the longer range communication of 868 MHz that allows nodes to directly connect to the root over longer distances. In this 25-node grid topology, however, it is still possible that a 868 MHz leaf node needs multiple hops to reach the root: as observed in Figure 12, there are some nodes that have one, two or three children, which indicates that the signal of those children to their parent was better compared to the signal of their link to the root. With 2.4 GHz communication that lacks such longer range capability, a packet typically has to traverse more hops to reach the root. For 2.4 GHz, there is also more variability in the lifetime of nodes with the same amount of children. For 868 MHz, we do not observe this effect. This is because the quality of the different links between the 2.4 GHz nodes differs in every experiment, resulting in a variable number of transmission cells and retransmissions that are necessary to deliver packets, which in turn also influences the energy consumption. Most 868 MHz nodes however are directly connected to the root with good link quality, resulting in almost no variability.

The results show that for a higher packet frequency, the average number of days a node lasts decreases, e.g., the average lifetime for 1 packet/s is 204 days compared to 487 days when having a frequency of 1 packet/h, for 2.4 GHz. The graph also shows that on average, the lifetime in a 868 MHz network is lower, because of the higher consumption values shown in Table 19. However, the results in Figure 11 showed that this does not necessarily hold for all nodes in a TSCH network.

## 6. Conclusions

In this paper, we propose a more accurate energy model for IEEE 802.15.4e TSCH using dual-band OpenMote hardware. The model differs from previous work in several ways. First, it includes an elaborate and up-to-date set of time slots and states and accurately models variable packet sizes. Second, we present state durations and energy consumption measurements for both the 868 MHz and 2.4 GHz frequency bands, using the CC1200 and CC2538 radio, respectively. We have experimentally verified the accuracy of the proposed model by comparing measured values of all time slots to their modeled counterpart. Furthermore, the energy consumption of a small-scale TSCH network was compared with its modeled consumption. For both the time slot comparison and the small-scale network experiment, the average error was less than 3%, including measurement inaccuracies and variations of the guard time. Using the measured energy slot consumption for both 868 MHz and 2.4 GHz communication, we also conducted several TSCH network simulations to observe the energy consumption effects for both frequency bands in an end-to-end context. We have also shown that the proposed model can accurately model all packet sizes, a feature absent in current TSCH energy consumption models, which only consider the maximum packet size. These results prove that our model is suitable to accurately predict the energy consumption of TSCH networks.

## Figures and Tables

**Table 1:** States in a TxDataRxAck slot.

State	CPU State	Radio State
TxDataOffsetStart	Active	Sleep
TxDataOffset	Sleep	Sleep
TxDataPrepare	Active	Idle
TxDataReady	Sleep	Idle
TxDataDelayStart	Active	Idle
TxDataDelay	Sleep	TX
TxDataStart	Active	TX
TxData	Sleep	TX
RxAckOffsetStart	Active	Sleep
RxAckOffset	Sleep	Sleep
RxAckPrepare	Active	Idle
RxAckReady	Sleep	Idle
RxAckListenStart	Active	Idle
RxAckListen	Sleep	Listen
RxAckStart	Active	RX
RxAck	Sleep	RX
TxProc	Active	Idle
Sleep	Sleep	Sleep

**Table 2:** States in a RxDataTxAck slot.

State	CPU State	Radio State
RxDataOffsetStart	Active	Sleep
RxDataOffset	Sleep	Sleep
RxDataPrepare	Active	Idle
RxDataReady	Sleep	Idle
RxDataListenStart	Active	Idle
RxDataListen	Sleep	Listen
RxDataStart	Active	RX
RxData	Sleep	RX
TxAckOffsetStart	Active	Idle
TxAckOffset	Sleep	Sleep
TxAckPrepare	Active	Idle
TxAckReady	Sleep	Idle
TxAckDelayStart	Active	Idle
TxAckDelay	Sleep	TX
TxAckStart	Active	TX
TxAck	Sleep	TX
RxProc	Active	Sleep
Sleep	Sleep	Sleep

**Table 3:** States in a TxData slot.

State	CPU State	Radio State
TxDataOffsetStart	Active	Sleep
TxDataOffset	Sleep	Sleep
TxDataPrepare	Active	Idle
TxDataReady	Sleep	Idle
TxDataDelayStart	Active	Idle
TxDataDelay	Sleep	TX
TxDataStart	Active	TX
TxData	Sleep	TX
TxProc	Active	Sleep
Sleep	Sleep	Sleep

**Table 4:** States in a RxData slot.

State	CPU State	Radio State
RxDataOffsetStart	Active	Sleep
RxDataOffset	Sleep	Sleep
RxDataPrepare	Active	Idle
RxDataReady	Sleep	Idle
RxDataListenStart	Active	Idle
RxDataListen	Sleep	Listen
RxDataStart	Active	RX
RxData	Sleep	RX
RxProc	Active	Idle
Sleep	Sleep	Sleep

**Table 5:** States in a RxIdle slot.

State	CPU State	Radio State
RxDataOffsetStart	Active	Sleep
RxDataOffset	Sleep	Sleep
RxDataPrepare	Active	Idle
RxDataReady	Sleep	Idle
RxDataListenStart	Active	Idle
RxDataListen	Sleep	Listen
RxProc	Active	Sleep
Sleep	Sleep	Sleep

**Table 6:** States in a Sleep slot.

State	CPU State	Radio State
SleepStart	Active	Sleep
Sleep	Sleep	Sleep

**Table 7:** States in a TxDataRxNoAck slot.

State	CPU State	Radio State
TxDataOffsetStart	Active	Sleep
TxDataOffset	Sleep	Sleep
TxDataPrepare	Active	Idle
TxDataReady	Sleep	Idle
TxDataDelayStart	Active	Idle
TxDataDelay	Sleep	TX
TxDataStart	Active	TX
TxData	Sleep	TX
RxAckOffsetStart	Active	Sleep
RxAckOffset	Sleep	Sleep
RxAckPrepare	Active	Idle
RxAckReady	Sleep	Idle
RxAckListenStart	Active	Idle
RxAckListen	Sleep	Listen
TxProc	Active	Sleep
Sleep	Sleep	Sleep

**Table 8:** State durations in the TxDataRxAck time slot with a total length of 15 ms and s being the packet size in bytes.

State	Duration (μs)	
	**CC2538**	**CC1200**
TxDataOffsetStart	105	105
TxDataOffset	1515	1454
TxDataPrepare	60+(s×0.875)	738+(s×8.152)
TxDataReady	1954−(s×0.875)	1276−(s×8.152)
TxDataDelayStart	17	58
TxDataDelay	349	369
TxDataStart	16	16
TxData	(3+s)×32−16	(3+s)×32−16
RxAckOffsetStart	32	75
RxAckOffset	3769	3116
RxAckPrepare	38	587
RxAckReady	267	328
RxAckListenStart	17	58
RxAckListen	483	442
RxAckStart	16	15
RxAck	880	881
TxProc	225	619
Sleep	5177−(s×32)	4783−(s×32)

**Table 9:** State durations in the Sleep time slot with a total length of 15 ms.

State	Duration (μs)	
	**CC2538**	**CC1200**
SleepStart	57	57
Sleep	14,943	14,943

**Table 10:** State durations in the RxIdle time slot with a total length of 15 ms.

State	Duration (μs)	
	**CC2538**	**CC1200**
RxDataOffsetStart	126	126
RxDataOffset	1567	1567
RxDataPrepare	38	676
RxDataReady	969	331
RxDataListenStart	17	58
RxDataListen	2583	2542
RxProc	25	118
Sleep	9675	9582

**Table 11:** State durations in the RxDataTxAck time slot with a total length of 15 ms and s being the packet size in bytes.

State	Duration (μs)	
	**CC2538**	**CC1200**
RxDataOffsetStart	126	126
RxDataOffset	1567	1567
RxDataPrepare	38	676
RxDataReady	969	331
RxDataListenStart	17	58
RxDataListen	1283	1242
RxDataStart	17	15
RxData	(3+s)×32−17	(3+s)×32−15
TxAckOffsetStart	126+(s×0.91)	362+(s×8.439)
TxAckOffset	3443−(s×0.91)	2810−(s×8.439)
TxAckPrepare	153	930
TxAckReady	518	77
TxAckDelayStart	17	58
TxAckDelay	349	369
TxAckStart	16	15
TxAck	880	881
RxProc	94	135
Sleep	5308−(s×32)	5267−(s×32)

**Table 12:** State durations in the TxData time slot with a total length of 15 ms and s being the packet size in bytes.

State	Duration (μs)	
	**CC2538**	**CC1200**
TxDataOffsetStart	105	105
TxDataOffset	1515	1454
TxDataPrepare	60+(s×0.875)	738+(s×8.152)
TxDataReady	1954−(s×0.875)	1276−(s×8.152)
TxDataDelayStart	17	58
TxDataDelay	349	369
TxDataStart	16	16
TxData	(3+s)×32−16	(3+s)×32−16
TxProc	72	109
Sleep	10,832 −(s×32)	10,795 −(s×32)

**Table 13:** State durations in the RxData time slot with a total length of 15 ms and s being the packet size in bytes.

State	Duration (μs)	
	**CC2538**	**CC1200**
RxDataOffsetStart	126	126
RxDataOffset	1567	1567
RxDataPrepare	38	676
RxDataReady	969	331
RxDataListenStart	17	58
RxDataListen	1283	1242
RxDataStart	17	15
RxData	(3+s)×32−17	(3+s)×32−15
RxProc	198+(s×0.91)	488+(s×8.439)
Sleep	10,706 −(s×31.09)	10,416 −(s×23.561)

**Table 14:** State durations in the TxDataRxNoAck time slot with a total length of 15 ms and s being the packet size in bytes.

State	Duration (μs)	
	**CC2538**	**CC1200**
TxDataOffsetStart	105	105
TxDataOffset	1515	1454
TxDataPrepare	60+(s×0.875)	738+(s×8.152)
TxDataReady	1954−(s×0.875)	1276−(s×8.152)
TxDataDelayStart	17	58
TxDataDelay	349	369
TxDataStart	16	16
TxData	(3+s)×32−16	(3+s)×32−16
RxAckOffsetStart	32	75
RxAckOffset	3769	3116
RxAckPrepare	38	587
RxAckReady	267	328
RxAckListenStart	17	58
RxAckListen	983	942
TxProc	44	137
Sleep	5754−(s×32)	5661−(s×32)

**Table 15:** Current drawn during different device states.

CPU State	Radio State	Consumption (mA)	
		**CC2538**	**CC1200**
Active	Sleep	13.97	15.06
Active	Idle	13.97	17.49
Active	Listen	31.14	40.13
Active	RX	26.94	50.63
Active	TX	31.47	54.26
Sleep (PM_NOACTION)	Sleep	10.06	11.42
Sleep (PM_NOACTION)	Idle	10.06	13.82
Sleep (PM_NOACTION)	Listen	27.18	36.18
Sleep (PM_NOACTION)	RX	23.16	46.73
Sleep (PM_NOACTION)	TX	27.55	50.24
Sleep (PM2)	Sleep	0.00156	0.27
Sleep (PM2)	Idle	0.00156	2.64

**Table 16:** Measured and calculated charge drawn for each slot type.

Slot Type	Measured (μs)		Calculated (μs)	
	CC2538	CC1200	CC2538	CC1200
TxDataRxAck	250.35	420.01	250.94	407.81
RxDataTxAck	253.2	432.09	251.32	417.2
TxData	229.8	360.2	230.13	357.12
RxData	235.1	373.55	228.72	362.12
RxIdle	197.4	245.2	196.35	240.98
Sleep	152.4	168.65	151.12	171.51
TxDataRxNoAck	246.95	395.65	246.79	384.94

**Table 17:** Measured and calculated charge drawn during a slot frame.

Mote Type	Measured (μC)		Calculated> (μC)	
	CC2538	CC1200	CC2538	CC1200
Leaf (Sleep)	7833.6	8698.05	7752.35	8816.48
Leaf (TxDataRxAck)	7910.1	8942.85	7852.17	9052.78
Relay	8086.05	9348.3	8002.81	9442.96

**Table 18:** Parameter configuration in the 6TiSCH simulator.

Parameter	868 MHz	2.4 GHz
Timeslot duration	15 ms	
Slot frame size	101 time slots	
No. of SHARED cells	1	
Inter-node distance	70 m	
No. of stable neighbors	1	
RPL parent set size	1	
Traffic period	5 s	
Traffic period variability	0.05	
EB period	10 s	
EB probability	0.15 s	
DIO period	30 s	
DIO probability	0.15	
OTF threshold	2	
OTF housekeeping period	10 s	
6top housekeeping	False	
TX power	0 dBm	
No. of channels	1	16
Stable RSSI	−83 dBm	−78 dBm

**Table 19:** Calculated charge drawn for each slot type, used in the simulator experiments.

Slot Type	Calculated (μC)	
	CC2538	CC1200
TxDataRxAck	106.45	275.61
RxDataTxAck	107.66	286.76
TxData	83.07	210.32
RxData	82.97	219.8
RxIdle	47.54	81.57
Sleep	0.82	0.89
TxDataRxNoAck	100.32	246.98
